# Motivational drivers for health professionals in a large quality improvement collaborative project in Brazil: a qualitative study

**DOI:** 10.1186/s12913-024-10678-w

**Published:** 2024-02-09

**Authors:** Eliane Pereira da Silva, Pedro Jesus Saturno-Hernández, Marise Reis de Freitas, Zenewton André da Silva Gama

**Affiliations:** 1 Department of Clinical Medicine, Federal University of Rio Grande do Norte, Natal, RN Brazil; 2 Graduate Program of Collective Health, Federal University of Rio Grande do Norte, Natal, RN Brazil; 3 National Institute of Public Health, Cuernavaca, Morelos México; 4 Department of Infectious Diseases, Federal University of Rio Grande do Norte, Natal, RN Brazil; 5 Department of Collective Health, Federal University of Rio Grande do Norte, Natal, RN Brazil

**Keywords:** Quality improvement, Healthcare quality, Collaborative projects, Motivation, Health professionals

## Abstract

**Background:**

The success of collaborative quality improvement (QI) projects in healthcare depends on the context and engagement of health teams; however, the factors that modulate teams’ motivation to participate in these projects are still unclear. The objective of the current study was to explore the barriers to and facilitators of motivation; the perspective was health professionals in a large project aiming to implement evidence-based infection prevention practices in intensive care units of Brazilian hospitals.

**Methods:**

This qualitative study was based on content analysis of semistructured in-depth interviews held with health professionals who participated in a collaborative QI project named “Improving patient safety on a large scale in Brazil”. In accordance with the principle of saturation, we selected a final sample of 12 hospitals located throughout the five regions of Brazil that have implemented QI; then, we conducted videoconference interviews with 28 health professionals from those hospitals. We encoded the interview data with NVivo software, and the interrelations among the data were assessed with the COM-B model.

**Results:**

The key barriers identified were belief that improvement increases workload, lack of knowledge about quality improvement, resistance to change, minimal involvement of physicians, lack of supplies, lack support from senior managers and work overload. The primary driver of motivation was tangible outcomes, as evidenced by a decrease in infections. Additionally, factors such as the active participation of senior managers, teamwork, learning in practice and understanding the reason for changes played significant roles in fostering motivation.

**Conclusion:**

The motivation of health professionals to participate in collaborative QI projects is driven by a variety of barriers and facilitators. The interactions between the senior manager, quality improvement teams, and healthcare professionals generate attitudes that modulate motivation. Thus, these aspects should be considered during the implementation of such projects. Future research could explore the cost-effectiveness of motivational approaches.

**Supplementary Information:**

The online version contains supplementary material available at 10.1186/s12913-024-10678-w.

**Table:** 

**Text box 1. Contributions to the literature**
• This study contributes to the understanding of the determinants of success in quality improvement projects in the context of middle- and low-income countries.
• The results add to the knowledge about the mechanisms involved in motivating professionals to actively participate in collaborative projects to improve healthcare.
• Theoretical models of implementation research that consider determinants of changes in health professionals’ behavior and efficiency in quality improvement can be refined in light of these results.
• The involvement of senior managers, performance of the quality improvement team, feedback on results, knowledge sharing, and teamwork are all found to motivate health professionals and thus must be considered during the design projects dedicated to the implementation of collaborative practices or quality improvement.

## Background

Improving access to quality healthcare has been a challenge for many countries [1, 2]. Advances made in regard to effective treatments and the control of infectious diseases have increased population survival; however, health systems still need to increase access to high-quality services [3]. Poor-quality healthcare is responsible for 8 million deaths per year, loss of quality of life, and approximately 6 trillion dollars of economic loss. For some diseases, the number of deaths associated with poor-quality healthcare is greater than that related to lack of access to treatment [4].

The quest for strategies to implement and assure quality in health services increased significantly in the 1990s. For example, in the context of primary healthcare in Portugal and Spain, the pioneer Iberian Program successfully implemented a strategy that was similar to the current collaborative model; it includes multisite projects and a structured problem-solving training model, and it reinforces internal commitment, professional leadership, and teamwork [5]. Health services have adapted new dynamic and interactive methods that were originally used in manufacturing industries (e.g., the Plan‒Do‒Study‒Act [PDSA] and Lean and Six-Sigma models) [6]. The Institute for Healthcare Improvement (IHI) introduced the collaborative model first in the USA and then subsequently in other countries using the science of improvement with a systemic vision, deep knowledge, knowledge of variations, and human engagement in quality improvement [7–9].

While the impact of collaborative QI projects in health systems is promising [10, 11], recent systematic reviews reveal results that are very heterogeneous in middle- and low-income countries [12, 13]. Health systems may need to not only update and disseminate best practices but also increase and apply knowledge about the underpinnings to develop a culture of quality [14] and the motivation to continuously improve the quality of healthcare [15]. Analyzing the drivers, barriers, and facilitators of improvement in the domains characterizing the context of health services may contribute to understanding the success or failure of collaborative QI projects [16, 17]. For instance, the Model for Understanding Success in Quality (MUSIQ) proposes considering factors related to the external environment, the entire organization (macrosystem), patient care units (microsystem), and the relationship between senior managers and QI teams [18]. These authors also described the relationship between capacity for improvement and motivation, which is directly linked to intervention results [18].

Motivation is agreed upon as a key factor for behavioral change; however, studies on this topic for quality improvement are scarce. A systematic review of the factors contributing to the results of collaborative quality improvement projects revealed gaps in understanding the mechanisms related to motivation and engagement [19]. The Capability, Opportunity, Motivation, and Behavior (COM-B) model used to improve evidence-based practice divides motivation into a reflexive (i.e., achieved by knowing and understanding the proposed behavior) and an automatic component (i.e., achieved by associative learning or innate disposition) [20].

Self-determination theory provides a multidimensional understanding of work motivation and its results. Intrinsic motivation is defined as innate, spontaneous, essential for cognitive and social development, and a source of pleasure throughout the life course. However, other factors also induce people to act. Extrinsic motivation is defined as engaging in a performance to achieve a result, and this motivation is influenced by factors ranging from external drivers to self-determined internal control [21, 22]. In addition, the satisfaction of innate needs (e.g., competence, autonomy, and interpersonal relationships) strongly drives motivation [23]. Understanding these complex mechanisms may be essential for supporting adherence to good practices.

Problems related to healthcare quality are plentiful in middle- and low-income countries; thus, a qualified and motivated workforce is needed to address these problems [24, 25]. Most countries need more qualified health professionals working in QI within a nonmotivational context. Previous research conducted in some middle- and low-income countries recommends evaluating and measuring motivation considering its context in boosting collaborative QI projects [26, 27].

The establishment of the Unified Health System in Brazil (SUS) has led to considerable advancements in recent years. However, despite progress, substantial challenges persist. In addition to concerns about per capita spending, notable issues include gaps in population coverage and deficiencies in quality metrics [28].

In 2018, Brazil invested millions of reais (local currency) in a pioneer national project directed toward collaborative quality improvement projects in healthcare in 116 hospitals all over the country [29, 30]; the primary outcomes consisted of reducing mortality and ventilator-associated pneumonia as well as central-line associated bloodstream infections and catheter-associated urinary tract infections. Other initiatives have been taken to analyze the implantation of primary healthcare in Brazil, especially to control diabetes and hypertension [31] and rare diseases occurring in Latin America [32].

However, until now, there has been a noticeable gap in aspects of motivation from a Brazilian health professional´s perspective with regard to participating in projects that improve healthcare. As in other countries, such a motivated workforce could drive and sustain engagement in quality improvement projects; this could result in the control of mortality rates and infectious diseases. Thus, this study aimed to understand the barriers to and facilitators of motivating health professionals to participate in a large quality improvement collaborative healthcare project in Brazil. The ultimate objective was to shed light on and analyze any possible modulatory effects of these barriers and facilitators on quality improvement. This study may have important implications for helping clinicians, microsystems, and governments work together while using better decision-making insights to construct a sustainable environment for planning and improving the quality of assistance within a health system.

## Methods

### Study design and context

This qualitative study was based on in-depth interviews with healthcare professionals working on a collaborative quality improvement project named “Improving patient safety on a large scale in Brazil” (PROADI-SUS) (30). The consolidated criteria for reporting qualitative research (COREQ) checklist was used. The project was conducted from January 2018 to December 2020 by the Brazilian Ministry of Health in partnership with hospitals within the Institutional Development Support Program of the Unified Health System (SUS) and the IHI. The aim was to implement prevention bundles to reduce healthcare-associated infections in intensive care units (ICUs), specifically ventilator-associated pneumonia, central-line associated bloodstream infections, and catheter-associated urinary tract infections.

### Participants

The initial collaborative project involved 116 public and private nonprofit hospitals that provided services to the SUS and were distributed throughout 24 of the 27 federative units of Brazil. In these hospitals, prevention bundles were implanted by the IHI and the Brazilian Health Minister from 2018 to 2020. Using the PROADI-SUS database, lists of hospitals, managers, and members of the quality improvement team at the hospital were consulted to help determine potential participants in the current barrier and facilitation study. Based on the available hospitals and according to the principle of saturation assessment, a final sample of 12 hospitals representing all five regions of the country was selected. The main inclusion criteria were based on (1) having an active quality improvement team at the hospital, (2) including at least one hospital in each of the Brazilian geographic regions, and (3) reaching the point of saturation for responses made by the interviewers. The exclusion criteria included hospitals where the quality improvement team was no longer active, had not responded after successive attempts to contact them by email or phone call, or declined participation spontaneously. The interviews were selected using purposive sampling and included leaders of quality improvement teams of certified and regulated healthcare professionals. Experienced healthcare professionals, such as nurses, physicians, physiotherapists, pharmacists, and nursing technicians, were able to participate only if they were active quality improvement teams. Alongside these graduated professionals, other health workers, such as nursing technicians, who worked at the hospital and were part of that team of quality improvement, were included. According to Brazilian law, nursing technicians are certified professionals who can manipulate patients in different hospital settings, including by introducing airway, urinary and venous catheters; therefore, they were included in this study.

Two hospitals declined to participate in the study because they no longer had the same improvement team working at the time of data collection. The study was approved by the research and ethics committee of the Onofre Lopes University Hospital of the Federal University of Rio Grande do Norte (no. 45047121.1.0000.5292), and each participant provided electronic informed consent before the interviews.

### Data collection

The data were collected between August 2021 and August 2022. A researcher with experience in qualitative research (EPS; woman, physician, and leader of the quality improvement team) performed the semi structured interviews. Participants were initially invited by email and then contacted by phone. The framework used for the interviews included previous points arising from preexisting collaborative literature on the quality of healthcare and a previous pilot study (data not included in this analysis). The interviews started with open-ended questions about motivation in the work environment, which were followed by questions focused on what influenced motivation and the barriers to and facilitators and maintenance of the project (see script in Additional File 1). Considering the restrictions imposed by the COVID-19 pandemic, interviews were conducted in a private session (lasting until 60 min) using Google Meet. Audio and video recordings were made of all interviews, which were then fully transcribed and returned to participants for comment and/or correction. The researcher had no formal relationship with the participants of the study. Only at the time of the interview were the participants known to the researcher; however, all the participants were made aware of the purpose and aims of the study at the first contact by either email or phone. No repeated interviews were conducted.

### Data analysis

The transcripts were examined using a content analysis approach [33]. Coding was performed after the interviews were read and reread by a member of the research team (EPS) to identify patterns and themes; the results were reviewed by another member of the research team (ZASG). NVivo software (QSR International Pty. Ltd.) was used as a tool to develop the coding and data management. Codes were assigned to represent quotes, and identified subcodes were integrated into the coding tree and categorized as barriers and facilitators (see supplementary Table S1, Additional File 1).

In addition, to gain a deep understanding of the interactions among the answers described as either barriers or facilitators, we used the MUSIQ and COM-B models as theoretical models to analyze any possible factors capable of changing behavior. We chose either prepublished themes from these models or other potentially relevant themes raised from private meetings. These models recognize that behavior in public or private-sphere organizations can change due to several factors.

## Results

### Characteristics of participants

Invitations were progressively sent to the selected hospitals, and interviews were scheduled. Professionals (mean length of time working at the same position = 11 years) from 12 hospitals were included in the sample. We interviewed 28 health professionals (16 nurses, 5 physicians, 3 physiotherapists, 2 pharmacists, and 2 nursing technicians). The interviews lasted between 20 and 53 min each. The hospitals represented all regions of Brazil (south = 3; southeast = 3; north = 2; northeast = 2; and midwest = 2); six were public hospitals, and six were private nonprofit hospitals related to the SUS. The mean number of hospital beds was 230.

### Categories of factors related to motivation

The barriers identified were belief that improvement increases workload, lack of knowledge about quality improvement, resistance to change, minimal involvement of physicians, lack of supplies, lack of support from senior managers and work overload. In contrast, the results achieved, the active participation of senior managers, teamwork, learning in practice and understanding the reason for changes were found to be facilitators of motivation. These themes were derived from quotes of interviews with health professionals (see supplementary Table S2, Additional File 1).

### Barriers

#### Belief that improvement increases workload

A strong belief was observed among healthcare professionals that projects and initiatives aimed at improving the quality of healthcare increase the workload. Professionals who already had a demanding work schedule felt uncomfortable and demotivated when asked to take on a new task in this regard.

“*What I see is that there is no continuity of service. People are not very interested since some changes require time. ‘Oh, we can discard all the diuresis in a single bucket since it is better, faster, and a one-way trip’. It takes work to do the right thing and change the routine*” (Interview 2, nurse, leader of a quality improvement team, public hospital).

#### Lack of knowledge about quality improvement

Systematic quality improvement is unusual in Brazilian hospitals. The Brazilian Ministry of Health has sought partnerships to improve health and allow professionals who participate in the initiatives to learn the quality improvement method. However, a dissemination strategy has not been established. In this study, we observed that few professionals had prior knowledge of the method used in collaboration. The project introduced the methodology to the leaders of the quality improvement teams; however, excessive tasks and work overload for the leaders in each hospital hindered the dissemination of that knowledge to a larger group of staff.

“*Education related to the knowledge of techniques is important; the education focuses on the improvement, socialization, and dispersion of this knowledge. If we are not careful to pass this daily, it ends up retained in only two or three people in a group*” (Interview 1, physician, member of a quality improvement team, nonprofit hospital).

#### Resistance to change

When the quality management team began implementing the quality project, the main aspect mentioned by the interviewees was resistance to change among some healthcare professionals; this resistance was described as a barrier to starting the project. Healthcare professionals were already working as health workers at the hospital unit before the implementation of the quality project. Normally, as they were already completely intimate with the clinical routine of that unit, these healthcare professionals revealed several factors that shaped a lack of motivation: lack of personal interest, financial incentives, empathy, or the time needed to fulfill tasks and measurement indicators; resistance to leaving one’s comfort zone; and presence of family in the ICU.

#### Minimal involvement of physicians

In multidisciplinary team organizations, nurses and physical therapists worked cohesively within quality improvement teams, whereas physicians were the least involved individuals, thereby demotivating other professionals.

“*We had difficulty with the medical team; the physicians always said everything was fine, but when we looked at the indicators, for all those indicators that we monitored, we could see that everything was not so good. The numbers do not lie”* (Interview 4, nurse, member of a quality improvement team, nonprofit hospital).

#### Lack of supplies

Another cause that negatively affected motivation according to the interviews was the discontinuity of supplies essential for adhering to prevention bundles, which was very discouraging for professionals in public hospitals. For example, the lack of a sterile transparent cover for a central venous access device implied nonadherence to the bundle; thus, 100% adherence could not be reached even when performing all other actions included in the bundle. In those hospitals, bureaucratic modification of the existing processes and improvements involving an immediate increase in costs (regardless of the amount) required commitment from those involved.

“*The main barrier is the discontinuity of the input supply, it is very irregular, often you and do not have”* (Interview 11, physician leader of the quality improvement team, public hospital).

#### Lack of support from senior managers

Senior managers induced a negative effect on motivation, depending on their actions. The commitment of managers was requested from the beginning of the project. It was demotivating when managers were not involved and did not participate in the rounds in the units but demanded results.

“*At the hospital where I was a leader, some managers, in a report presentation meeting, wanted the result to magically happen without a collective effort, without at least a small portion of flexibility in human resources.*” (Interview 1, physician, leader of the quality improvement team, non-profit hospital).

About a quarter of the studied hospitals faced management changes. These changes caused insecurity in the quality improvement and healthcare teams regarding support for the ongoing project. Having a manager without health training was another barrier to motivation, especially for the leaders of the quality improvement teams. The team spent a great time convincing them about the importance of supplies for maintaining good practices since some implied increased costs.

“*We have senior managers that are not from the health area. They did not understand the importance of the project, they did not understand the need to change some processes.”* (Interview 21, nurse, leader of the quality improvement team, non-profit hospital).

#### Work overload

This barrier was related to the fact that some hospitals, especially those located outside of large urban centers, do not have an organized structure for quality management. Quality improvement teams were formed for the collaborative project and included professionals from the patient safety center, the hospital infection control committee, and the ICU. In this sense, these professionals still performed other healthcare or management activities and thus gained an additional role in the collaborative quality improvement project. Thus, professionals on the quality improvement team frequently reported experiencing work overload and a lack of work time allowed for the project. Their participation was considered extra work; thus, the majority of the time, being involved in the quality improvement project of the microsystem was considered a demotivating aspect.

“*The leader of the quality improvement team who worked with us had multiple roles. So, not everything that was proposed in the project was implemented”* (Interview 22, nursing technician, member of a healthcare team, nonprofit hospital).

The tasks of the quality improvement team were described as sometimes “arduous”, especially when it was necessary to demand engagement from professionals resistant to changes. Although rare, strained relationships were reported, especially involving experienced professionals and those linked to public health institutions (i.e., with stable employment).

### Facilitators

#### The results achieved

A reduction in infections was the main positive factor for motivation in collaborative quality improvement projects. We observed great motivation in healthcare professionals after the first reductions in incidence rates. Additionally, patient recovery and the absence of infections during ICU stays increased the sense of accomplishment related to quality healthcare.

“*Showing the results to the team—that patient had been cured, improved, returned to society, to their family—showing this result of patients being made well to the team was an important factor in their motivation*” (Interview 18, nurse, member of a healthcare team, nonprofit hospital).

Celebrating the results encouraged by the quality improvement collaborative project also positively motivated healthcare professionals.

“*The results were great motivators. When we achieved the results of reducing ventilator-associated pneumonia, and the team saw that it was possible, it was a great motivator*” (Interview 17, physical therapist, member of a healthcare team, public hospital).

#### The active participation of senior managers

Senior managers reportedly induced a motivating effect (facilitator) when they were present in the units, listening to professionals and reinforcing and committing to an institutional policy of continuous improvement, as observed in the following comment made by an interviewed professional. In this sense, hospital managers played an essential role in dictating the pace of collaborative quality improvement projects and favoring the motivation for change.

“*It always made a difference when our manager went to the ICU, talked to collaborators, and called teams in front of the monitoring board. People felt valued. ‘Look, they are looking at us here; they want to understand our difficulties’. The rounds made by senior managers were always very positive”* (Interview 7, pharmacist, leader of a quality improvement team, nonprofit hospital).

#### Teamwork

Facilitators of teamwork and the division of healthcare professionals into quality improvement teams were also described (each one of which was responsible for a bundle). They were described as very positive and motivated by all professionals. The ability of creativity to promote the bundling and conducting of the PDSA approach to test changes increased the commitment level and sense of belonging to the project. When one professional was cheerless, the other was positive, thereby maintaining the team’s balance and motivation.

“*What made it easier was the unity of the team. What I did in the project was to include the whole team and assemble smaller teams. We made identification codes, i.e., a yellow one for the urinary infection team and a red one for the bloodstream infection team. That motivated them, and every month, they presented the results in front of the organizational board*” (Interview 13, nurse, member of a quality improvement team, public hospital).

#### Learning in practice and understanding the reason for changes

We observed that participation in a collaborative quality improvement project added value to healthcare professionals at all educational levels and was a facilitator of motivation to participate in the QI project. Even those with experience in quality improvement added new experiences to their previous knowledge, thereby expanding the scope of action.

“*The good thing about the project was that it helped us to search for the technical part of how to do it; we wanted to improve, but we had no method to do that*” (Interview 20, pharmacist, member of the healthcare team, nonprofit hospital).

In general, healthcare professionals without experience in quality improvement methods incorporated a new concept of action in the quality management area of healthcare. They reported that the experiences provided by the project changed them. The benefits that may be achieved by saving lives when investing in quality were a top motivator for leaders and teams.

They also facilitated motivation to understanding the importance of making small adjustments in work processes by the healthcare team. Such actions changed the behavior of the team, which in turn adjusted the process of work, incorporated prevention bundles, and explained each care action performed. Even initially resistant professionals allegedly changed their practices after undergoing this educational process.

“*When the healthcare team understood why we were pursuing the hygiene of the connectors and elevating the headboards, we started to see motivation; we started to receive questions about how the infections were going and how we were providing oral hygiene*” (Interview 1, physician, member of a quality improvement team, nonprofit hospital).

### Theorical framework for understanding the agent modulators of the motivation of professionals in collaborative quality improvement projects

**Fig. 1:**
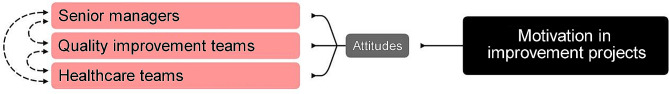
Theorical framework of understanding the agent modulators of the motivation of professionals in the quality improvement collaborative project

From our observations of the interviewees, we noted an interrelationship among the three main agents involved in the motivation to participate in quality improvement collaborative projects, namely, senior managers, quality improvement teams, and healthcare teams. Any of these agents may start changes, which may arise from institutional needs, risk situations, or perceived opportunities for improvement. Government policies and risk management may influence senior managers and motivate change to meet regulatory requirements. The quality improvement and healthcare teams start the motivation based on internal demands of the macro- and microsystems. Additionally, the quality improvement team connects senior managers with the healthcare team, provides knowledge to healthcare professionals, and encourages motivation. Figure 1 summarizes the theoretical framework of the interrelationship of our data, in which the attitudes of those involved result in the motivation to participate in collaborative quality improvement projects.

## Discussion

This study pioneered the analysis of the motivation behind collaborative QI projects in Brazil; a middle- and low-income countries with cultural and social diversity, lack of tradition in large-scale quality improvement collaborative projects, and need to improve its health systems. The study sample included hospitals from all regions of the country enrolled in a project that achieved successful results. The findings provide a perspective for future studies to test these hypotheses. Additionally, funders and managers of collaborative quality improvement projects may consider specific interventions for the motivation factors discussed in this study.

The quality collaborative improvement project was an interesting case study for building a model of understanding the motivation for improving the quality of assistance. The hospitals had several contexts, varying from organizations of excellence with international accreditation and professionals trained in quality improvement to those with difficulties in basic quality improvement processes [34]. The 12 hospitals represented such diversity because they were from different regions in Brazil and included both public and private nonprofit hospitals. Despite the heterogeneity of the hospitals involved [35], the project successfully achieved a 55% reduction in healthcare-associated infections, saving 2,687 lives [30].

Considering that Brazil has continental dimensions, the regional and institutional characteristics of hospitals determine the dynamic contexts influenced by national and state policies, generating forces that interfere with health organizations and microsystems. The activities conducted in the microsystem and the performance of quality improvement teams are directly responsible for the results of collaborative projects [18]. However, even with the heterogeneity of hospitals and recently trained quality improvement teams, the potential for amplification and sustainability was observed for the achieved improvements.

Different actors were interested in participating in the project since it was a voluntary membership project supported by the Brazilian Ministry of Health and organized by national and international institutions renowned for quality management. The initiative came from professionals who worked in quality units, hospital infection commissions, and hospital management. The opportunity to be part of a learning group in an area where most hospitals need expertise was a great motivator for visionary managers and healthcare professionals [36].

According to our analysis, senior managers had either positive or negative impacts. Motivation was observed when the manager assumed a participative posture and left his or her office to walk through the hospital and listen to people. In contrast, when the manager was absent, the team doubted the institutional commitment to the project and related improvements. Although those involved in healthcare have a duty to recognize the need to improve the quality of care, the sustainability of improvements will depend on institutional policy and managers and reflect their actions in terms of the motivation and well-being of professionals [37, 38]. This knowledge is essential for supporting strategies for healthcare services facing barriers, such as those determined from a quality improvement project based on gestational syphilis in Rio de Janeiro [39]. This project faced difficulties in motivating professionals and achieving a positive effect due to an unfavorable context in the participating services [39].

The quality improvement team created a motivational atmosphere around the collaborative quality improvement project. Although not all professionals had training in the area, the teams were naturally interested in the issue of quality improvement and extremely motivated by the opportunity to improve hospital quality. The quality improvement teams received a structured project and were trained in the model for improvement methods in face-to-face meetings. A great challenge was encouraging healthcare professionals to maintain their motivation. In this group, we found resilient health professionals with autonomic motivation who were responsible for the progress of the project. Additionally, they overcame barriers, such as extended working hours, excessive bureaucracy, the need to involve healthcare professionals from all shifts in changes, and the patience required to encourage teamwork. The positive outcomes of and compensation for this team involved providing feedback on the positive results and celebrating the results together.

The healthcare professionals had insufficient knowledge of quality improvement but plenty of clinical experience. These professionals represent the largest labor contingent and are closest to the patients; additionally, they conduct certain actions and must engage in good practices to obtain results. Additionally, they respond with either adherence or resistance when faced with challenges related to improvements aiming to reduce infections [40]. Healthcare professionals were convinced of the knowledge and examples of leaders of quality improvement teams. The great motivation for these professionals was the result of their work in preventing infections and recovering the patient.

Improving healthcare in microsystems through patient-centered care and better interrelationship among professionals can lead to integrated, safe, and quality care [41]. Additionally, multidisciplinary teamwork encourages positive motivation. However, herein, the adherence of physicians to prevention bundles was found to require the intervention of leaders of quality improvement teams since some indicators depended on their presence in discussions and decisions. Several factors, such as medical training, personal interest, and work overload, were responsible for excluding physicians from the quality and patient safety guidelines. Similarly, the noninvolvement of physicians in the quality process within the microsystem was also relevant in an evaluation of contextual factors for the success of accreditation in Colombian hospitals, suggesting that this reality is not exclusive to Brazil [42].

Motivation is responsible for engagement, and the work environment may positively support motivation, promote growth, learning, autonomy, and competence, and it may positively influence intrinsic and extrinsic motivation [43, 44]. The design of quality improvement collaborative projects provides opportunities to improve motivation in all types of contexts concerning the resources available.

Based on the theory of the COM-B model, which defines the “behavior changes wheel” [20] as starting with capability, followed by motivation and opportunity, we observed that motivation influences behavior change by acting as a barrier to facilitators and defining the sustainability and organizational culture for quality improvement. Capability is considered a personal (psychological or physical) attribute that enables individuals to engage in the activity concerned. Opportunity is an environmental, organizational, and system-level attribute. Both capability and opportunity can either drive the behavior facilitating it or not. Motivation is defined as a set of mental processes that energize and direct behavior. These three forces interact to generate behavior from an organizational perspective.

Motivation in self-determination theory varies from controlled to autonomous motivation [22, 45]. Additionally, health professionals with high levels of intrinsic motivation, which is autonomous and innate to the individual, were observed in all hospitals. Positive emotions and a sense of self-worth from the collaborative environment (i.e., introjected regulation) motivated some health professionals, while others were motivated by purposes of the project resembling their own purposes (i.e., identified regulation). In addition, some health professionals were very comfortable with the changes because they felt that they were part of the project and had the same values.

The reflexive motivation in the COM-B model may be similar to the introjected and identified regulation in self-determination theory, which requires reflection and cognitive capacity for decision-making. Therefore, positive support from senior managers, the presence of a quality improvement team, and institutional policy may act as reflexive motivations. Automatic motivation is related to emotions and impulses and uses associative links according to the principles of contiguity and similarity, thereby modulating other autonomous mechanisms [46]. Thus, active participation and the understanding of changes and their positive results activate autonomous mechanisms of pleasure and satisfaction, which are related to improving the quality of healthcare [45].

By applying these principles, deepening the theoretical interactions between the coding of interviewers and the descriptions provided by the subjects of study and merging them into the context of the COM-B model [20], we found that, according to quality improvement, the collaborative project acted on three major pillars of behavioral change, namely, influencing motivation (the study focus), strengthening the capability for improvement, and providing government initiative with the offer of the project as an opportunity. After separating factors related to motivation, we assumed that the factors related to senior managers and the quality improvement team were reflective since they were external stimuli used to motivate healthcare professionals to participate in the project. Automatic factors motivate healthcare professionals individually based on a personal understanding of the reason for changes. Participating in the change tests, teamwork, and achieving results induced personal satisfaction.

**Fig. 2:**
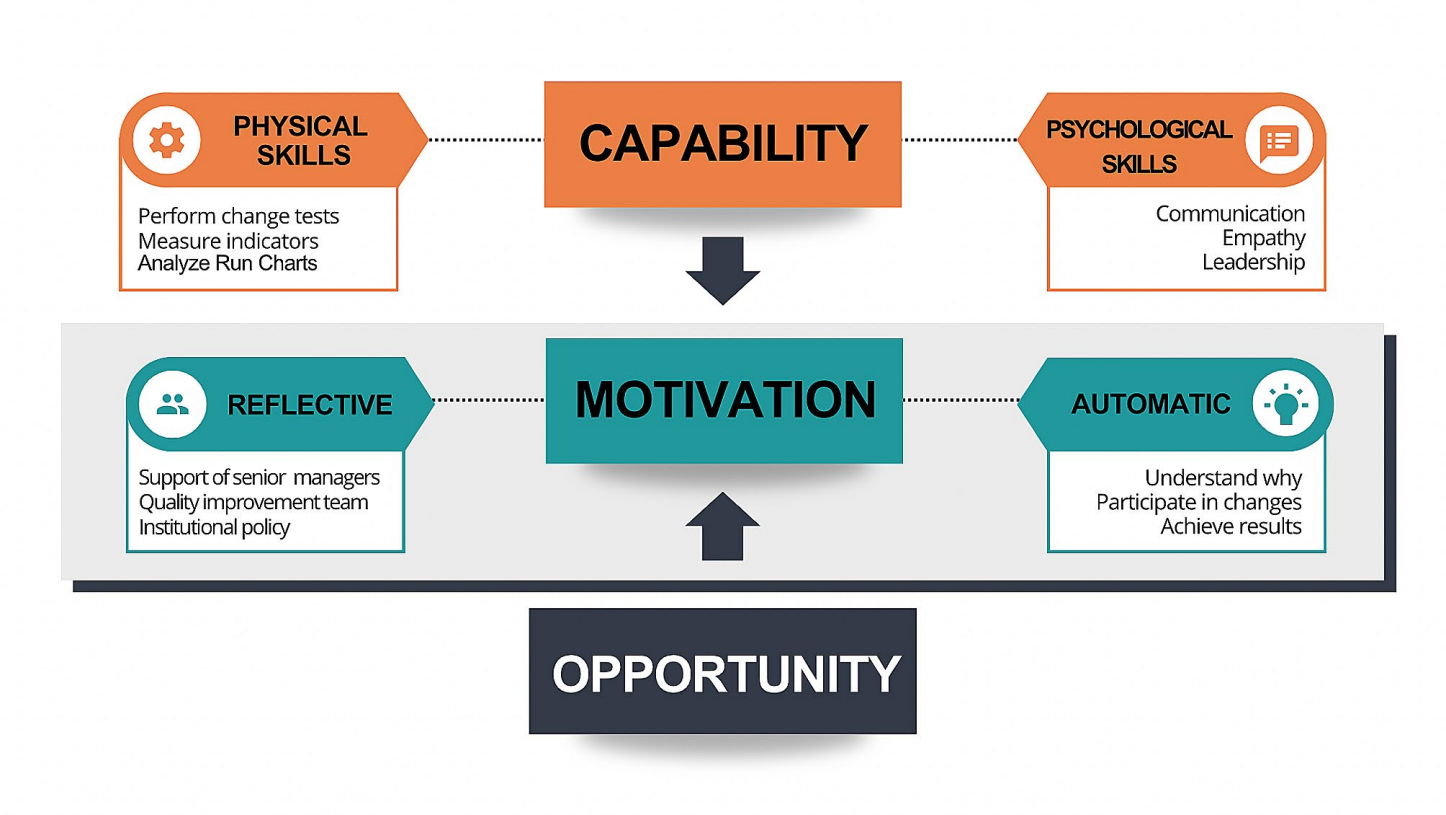
Potential interactions within the Capability, Opportunity, Motivation and Behavior (COM-B) model that could support collaborative quality improvement projects in hospitals. Adapted from Ref 20. Capability is defined an individual’s ability (psychological or physical capability) together with the available opportunity (an attribute of the environmental system and thus outside of the individual) that facilitates it. Motivation is defined as all brain processes that energize and direct one’s behavior. There is a distinguishing topic related to motivation between reflexive and automatic processes. Reflexive motivation involves evaluation and planning, including organizational motivation. According to our data, the support of managers, the QI team and institutional policy are the reflexive drivers of motivation. Automatic motivation involves instinctive, habitual, and affective processes. To the best of our knowledge, understanding why, participating in changes and achieving results are considered the main forces

The project strengthened the capability for improvement by developing physical or technical skills, such as performing PDSA, running and analyzing trend charts, implementing bundles, following a driver diagram, and measuring indicators. Many psychological and nontechnical skills were also improved (e.g., teamwork, leadership, empathy, negotiation, communication, and knowledge and team management), which was important for implementing changes and achieving goals. Additionally, some skills in this study are part of the skills pyramid for improvement obtained from the Health Foundation, which we suggest in Fig. 2 [34].

The quality improvement collaborative project achieved positive results even with difficulties related to the motivation of health professionals. Although data cannot assure that hospitals with motivated professionals are more likely to achieve goals, the achievement of such goals reflects a positive balance in motivation [47]. In a quality improvement collaborative project from the United Kingdom, a sense of personal achievement, priorities of organizations, engagement of multidisciplinary team members, and support of senior managers were found to be related to motivation [48]. Thus, motivation impacts success even in developed countries with a tradition of conducting collaborative quality improvement projects.

Spending resources on quality improvement should be considered an optimization of resources since having an organized and qualified quality improvement team impacts the motivation, capability, and sustainability of improvements [49]. Herein, it was found that hospitals that had trained professionals or learned from the project and created a permanent quality improvement team could sustain the implementation of good practices and monitor indicators. Additionally, achieving quality goals not only saved lives but also saved public resources, which need to be optimized since Brazil has chronic underfunding in the SUS.

We must assume that our data have several limitations. First, the generalization of our findings must be performed carefully. For instance, the researchers had access to only 10.2% of the 116 hospitals that developed the initial project of quality improvement for the control of infections. Thus, we assume the limited transferability of our findings. Second, most hospitals no longer had the same QI members at the time of data collection; furthermore, some of them had urgently restructured their healthcare professionals and attendance times because of the COVID-19 pandemic. This approach limited the representativeness of our data. However, our analyses showed a good level of saturation among the interviewees. Approximately 90% of the responses were focused on the same themes given by the 12 participating hospitals. Thus, it is possible that even if the number of interviews had been larger, the data would not have been expressively different enough to compromise the results. On the other hand, during the COVID-19 pandemic, most of the personnel in hospitals who participated in QI were highly trained in terms of infection control and could thus help hospitals and staff members reinforce strategies to address the pandemic. This represented a differential aspect of motivation to that provided by other healthcare workers. Second, due to the redirection of the workforce to COVID-19, most hospitals did not have access to individual outcome indicators related to the initial project objectives. Even though our initial aim was not to analyze infection outcome indicators, these data were described by interviewers to monitor progress and thus could be an important aspect of barriers and facilitators to QI. Therefore, we had only data from an individual perspective of healthcare professionals.

The data were collected during the COVID-19 pandemic, and interviews were conducted remotely eight months after the end of the project due to delays in approval from the research ethics committees of various states. Most ethics committees directed their efforts to accept only projects involving COVID-19 during 2020 and 2021, i.e., the first meaningful year of infection. Although this interview latency might have led to a memory bias, related memories may have endured since the project impacted the health professionals. Finally, even though professional quality teams have been well trained to manage infections and control them—these professionals have provided not only technical and emotional support to make decisions but also encouragement, scientific and technical expertise, and clinical training to other health workers and hospitals per se—the high demand for COVID-19 has increased anxiety, depression, stress, and burnout levels among all health professionals. In this sense, we assume that, in 2021, which represented the worst wave of COVID-19 infections in Brazil, all the interviewed professionals could have experienced some degree of labor, social and emotional symptoms related to the pandemic. As this theme is very sensible and includes several multifactorial aspects of motivation, the inherent motivation at the individual level being affected by any of the abovementioned symptoms could have interfered with perceptions of barriers or facilitators during the data collection period during the pandemic phase. Thus, we must consider these aspects when interpreting our data.

## Conclusion

The belief that improvement increases workload, lack of knowledge about quality improvement, resistance to change, minimal involvement of physicians, lack of supplies, lack of support from senior managers and work overload were the factors that most contributed as either barriers. Conversely, the results achieved, the active participation of senior managers, teamwork, learning in practice and understanding the reason for changes were facilitators of motivation in the collaborative quality improvement project. Thus, encouraging teamwork, providing ongoing education, supporting task management, and implementing an institutional policy of continuous improvement can assist individuals in overcoming resistance to change, reducing barriers, and enhancing facilitators to improve the outcomes of collaborative quality improvement projects. This study reinforced the importance of customizing large projects involving modulators of motivation, intervening in factors identified as barriers and facilitators, and verifying interactions in the microsystem to ensure maximum motivation during collaborative projects. Theoretical models of implementation research that consider determinants of changes in health professionals’ behavior and efficiency in quality improvement can be refined when considering these results. Future studies can analyze the importance of each of the barriers and facilitators found in this study by quantifying their importance. This may indicate the most efficient way to stimulate motivation. Furthermore, our results take an essential step towards the construction of instruments for measuring motivation of health professionals during improvement projects. Another possibility is to apply mixed methods of implementation research that study the weight of these factors for project success and explore the cost-effectiveness of motivational approaches.

### Electronic supplementary material

Below is the link to the electronic supplementary material.

Supplementary Material 1

Supplementary Material 2

## Data Availability

The datasets used and/or analyzed during the current study are available from the corresponding author on reasonable request.

## Abbreviations

IHI: Institute for Healthcare Improvement
MUSIQ: Model for Understanding Success in Quality
Capability: Opportunity,Motivation,and Behavior,COM-B
SUS: Unified Health System
ICUs: Intensive care units
PDSA: Plan‒Do‒Study–Act
