# Supplementary material for: Motivational drivers for health professionals in a large quality improvement collaborative project in Brazil: a qualitative study
Source: BMC Health Serv Res. 2024 Feb 9;24:183. doi: 10.1186/s12913-024-10678-w (PMC10854114; doi:10.1186/s12913-024-10678-w)
Supplement: Supplementary file 1 — Supplementary Material 1 [file 12913_2024_10678_MOESM1_ESM.docx]

**Additional File 1**

**Script of the in-depth interview**

Objective: To analyze barriers and facilitators to motivation of health professionals in quality improvement collaborative projects for healthcare.

I) PRESENTATION

1) First, thank the presence of the guest at the meeting.

2) Presentation of the researcher:

Say your name and highlight that you are a member of a Ph.D. study on quality improvement of health services of the Postgraduate Program in Collective Health. The aim of the study is to improve the quality of care by identifying barriers and facilitators to motivation in quality improvement.

3) Explain what we want: to know the opinion of healthcare professionals who participated in the quality improvement collaborative project about their motivation to participate.

4) We will send a link to a brief questionnaire to collect some information from guests.

II) SCRIPT OF THE IN-DEPTH INTERVIEW

Encourage the guest to remember their participation in the quality improvement collaborative project, asking what they remember about this experience. The interview will be preferably divided into three phases: beginning of the project, project development, and end of the project. The order of questions will be as follows:

BEGINNING OF THE PROJECT

1) Tell us about yourself and your work at the hospital.

2) What do you remember and how did you feel about the beginning of the project in your unit?

PROJECT DEVELOPMENT

3) What kept you and the team motivated during the project development?

4) What were the barriers to motivation? What demotivated you?

5) What was the role of the leadership in your enthusiasm to participate in the project? Consider everyone who influenced the team responsible for the project.

END OF THE PROJECT

6) How was the motivation at the end of the project?

7) How would you describe the characteristics/skills of the most motivated people in your team?

8) If you were responsible for the project, if you "commanded", what would you have done differently?

**Table S1** Coding tree of the thematic analysis from interviews with health professionals of the quality improvement collaborative project.

| **Minor codes** | **Major codes** | **Category** |
| --- | --- | --- |
| Lack of support from senior management | Health institution management | Barrier |
| Lack of supplies |  |  |
| Resistance to change presented by professionals | Improvement team challenges |  |
| Perform multiple functions |  |  |
| Belief that improvement increases workload | Assistance professionals (microsystem) |  |
| Minimal involvement of physicians |  |  |
| Work overload |  |  |
| Lack of knowledge about quality improvement | Continuing education |  |
| Active participation of senior managers | Health institution management | Facilitators |
| Listen to care professionals |  |  |
| Understanding the reason for changes | Assistance professionals (microsystem) |  |
| Teamwork |  |  |
| Participate in change testing |  |  |
| Learning in practice | Continuing education |  |
| Celebrate small results | Feedback |  |
| Tangible outcomes |  |  |

**Table S2** Illustrative quotes from interviews with health professionals and main barriers and facilitators of motivation to engage to quality improvement collaborative project.

| **N.** | **Quotes** |
| --- | --- |
| **Barriers** | |
| **Theme**: **belief that improvement increases workload** | |
| 1 | “What I see is that there is no continuity of service. People are not very interested since some changes require time. ‘Oh, we can discard all the diuresis in a single bucket since it is better, faster, and a one-way trip.’, it takes work to do the right thing and change the routine.” (Interview 2, nurse, leader of the quality improvement team, public hospital). |
| 2 | “Some understood the project as just one more thing to do or someone who said it was more service, more work” (Interview 3, nurse, member of a quality improvement team, nonprofit hospital). |
| **Theme**: **lack of knowledge about quality improvement** | |
| 3 | “We started not putting people from the ICU as project leaders. The manager of the hospital infection control committee ended up as the leader, and I, from the quality sector, as administrative support. Initially, this hampered the project because the ICU did not understand the project as their own to embrace it from the beginning, even participating in all the meetings.” (Interview 4, nurse, member of the quality improvement team, non-profit hospital). |
| 4 | “During the project, the quality sector was created, which did not exist before the project.” (Interview 8, physician, member of the quality improvement team, non-profit hospital). |
| 5 | “It was by chance, we signed up not knowing well what it would be, we ended up joining, and it was a very important cutoff point in the hospital. Until then, we did not know about the improvement method, I had already heard about the Plan-Do-Study-Act (PDSA) but not in-depth as we had in the project. All the learning, workshops, and exchange opportunities, I think it was a game changer for us.” (Interview 7, pharmacist, leader of the quality improvement team, non-profit hospital). |
| 6 | “We did not have many indicators, we used infection indicators, but process indicators and PDSA did not exist. Evaluating the elevation rate of the headboard at 45º, how they were cleaning the connectors, these were things that we could not even imagine how it could be done.” (Interview 8, physician, member of the quality improvement team, non-profit hospital). |
| **Theme: resistance to change** | |
| 7 | “Any change always met resistance, when we passed on to the technicians what we had decided, they always were resistant, they said that it was one more thing to do, one more piece of paper to write on, but as the days went by, people started to do it, it became routine, they did it without complaining.” (Interview 23, nurse, member of the healthcare team, non-profit hospital). |
| 8 | “I think that younger teams may engage more. As we get old, we become more resistant to change.” (Interview 12, nurse, member of the quality improvement team, public hospital). |
| 9 | “Every change cause discomfort. For example, the early removal of devices. We have experienced people in the adult ICU who have always thought that ICU is synonymous with device.” (Interview 15, nurse, member of the quality improvement team, public hospital). |
| 10 | “Some people stood out, we had a very united team, but of course, some people were resistant. In general, the experienced ones say, ‘we have always done it this way, why does it have to change now?’. Always someone on each team say, ‘let us change, let us do it differently, stop, let us do it differently.’, always someone counterbalanced it.” (Interview 21, nurse, leader of the quality improvement team, non-profit hospital). |
| 11 | “We had great difficulty engaging the night team, not only because they did not want to engage but because of the difficulty of being present at night and sharing information like during the day. The difference is that it is two hospitals with morning, afternoon, and night shifts.” (Interview 4, nurse, member of the quality improvement team, non-profit hospital). |
| **Theme: minimal involvement of physicians** | |
| 12 | “We had difficulty with the medical team; the physicians always said everything was fine, but when we looked at the indicators, for all those indicators that we monitored, we could see that everything was not so good. The numbers do not lie” (Interview 4, nurse, member of a quality improvement team, nonprofit hospital). |
| 13 | “We had difficulty accepting it from the medical side, even from the medical coordination itself, we had resistance so that the project could be accepted and so that we could run the PDSAs” (Interview 4, nurse, member of the quality improvement team, non-profit hospital). |
| **Theme: lack of supplies** | |
| 14 | “The main barrier is the discontinuity of the input supply, it is very irregular, often you and do not have” (Interview 11, physician leader of the quality improvement team, public hospital). |
| 15 | “What discourages us is that it's a public hospital, so from time to time we have a lack of supplies, a lack of transparent dressings, for example, pressure injury protection, sometimes we don't have fatty acids or nystatin ointment. What is discouraging to this day is the lack of inputs” (Interview 15, nurse, member of the quality improvement team, public hospital). |
| **Theme: lack of support from senior managers** | |
| 16 | “Support was more effective from coordinators than senior managers. During the project, we had five different managers, and the discontinuity of services was a characteristic factor in the transitions.” (Interview 6, nurse, member of the healthcare team, public hospital). |
| 17 | “The biggest barrier we had here was the hospital directors. We have senior managers that are not from the health area. They did not understand the importance of the project, they did not understand the need to change some processes. Sometimes during the project, we almost had to abandon it because we needed to sign the contract, he did not understand: ‘oh, why are we doing this? What are we going gain with this?’.” (Interview 21, nurse, leader of the quality improvement team, non-profit hospital). |
| 18 | “One barrier is the authorities in the hospitals. Senior management often has the role of a manager, but they do not experience the daily living of patients, they demand results and often do not understand that a visit by senior managers is very important within this context, being able to schedule these visits is not simple.” (Interview 1, physician, leader of the quality improvement team, non-profit hospital). |
| **Theme: work overload** | |
| 19 | “If it is not someone who is fully into it, maybe they already have other things to do. They put a person to collect the indicators but did not provide time, they already had other activities.” (Interview 12, nurse, member of the quality improvement team, public hospital). |
| 20 | “I had a lot to do, I had difficulty trying to teach them what I learned, this was very bad because I knew many things, but I did not have time to teach them. We did not have more hours, I was too overloaded to teach each one by talking, so that was a difficulty at the beginning to make people understand the project.” (Interview 13, nurse, leader of the quality improvement team, public hospital). |
| **Facilitators** | |
| **Theme: the results achieved** | |
| 21 | “That logic of celebrating small victories, perhaps of the project as a whole, is the main change we have in culture because, in general, people learn to celebrate big things.” (Interview 1, physician, leader of the quality improvement team, non-profit hospital) |
| 22 | “Toasting, celebrating the gains, this is something that we saw that people in the sector like, they like to feel, they like us to tell them that they did the service and because of that, we got a good result.” (Interview 20, pharmacist, member of the healthcare team, non-profit hospital). |
| 23 | “The result made them recognize their work, they said ‘look, we reduced it today, we are reducing it.’. That everyday reminder, the bundles that we learned to prevent ventilator-associated pneumonia, urinary infection, they saw the results, which was the reduction of infection and mortality.” (Interview 13, nurse, leader of the quality improvement team, public hospital). |
| 24 | “Showing the result to the team, that the patient was cured, improved, returned to society, to the family, showing this result to the team and the patient being well is an important factor in motivation.” (Interview 18, nurse, member of the healthcare team, non-profit hospital). |
| 25 | “When we started working with them on how the project reflected on patient safety, when they started to see the rates reducing, when we were able to see, ‘oh my God, there was no ventilator-associated pneumonia.’ then several people celebrated together. I think that was the great motivator, and it boosted engagement, seeing that it was possible to achieve.” (Interview 7, pharmacist, leader of the quality improvement team, non-profit hospital). |
| 26 | “So, working with quality improvement, we see that we need to walk a little to have a result, but they come, and we can prove that it is the best, we can prove that the improvement happened through everything that we harvested.” (Interview 3, nurse, member of the quality improvement team, non-profit hospital). |
| **Theme: the active participation of senior managers** | |
| 27 | “The meetings with senior managers in the ICU who listened to the employees were very well received by the team, I think this was also a motivational factor.” (Interview 4, nurse, member of the quality improvement team, non-profit hospital). |
| 28 | “The positive point is that, at least during the project, we had the involvement of senior managers, and I think this demonstrated a charge, people felt charged and obligated themselves to do the right thing.” (Interview 13, nurse, leader of the quality improvement team, public hospital). |
| 29 | “The call of the project for senior manager, coordinator, and director, I think this helps a lot, the role of the hospital is to engage in the project and not think that this is only one thing from the intensive care unit (ICU). Ask ‘so, how is your project? Improved or not?’. The project gains an institutional status.” (Interview 8, physician, member of the quality improvement team, non-profit hospital). |
| 30 | “I had difficulties with senior managers, every time I got a manager to go to the ICU, the team was motivated, although I knew they went due to my insistence. I noticed that the role of senior managers is essential, the presence, care, really being present, listening to the employee, they will not ask for a raise or million things, having recognition was fantastic.” (Interview 3, nurse, member of the quality improvement team, non-profit hospital). |
| **Theme: teamwork** | |
| 31 | “The leader visited us daily to talk, listen, observe, and replan on the daily issues, problems that appeared at the moment, asking ‘what do you suggest? What can we do differently?’. Doing the different is where the differential is.” (Interview 22, nursing technician, member of the healthcare team, non-profit hospital). |
| 32 | “Every tool I implemented had their participation in elaboration, nothing was top-down. ‘Starting tomorrow, you will have to do this.’, I did not do any of that, what I did, I brought it, explained the importance, listened what was their opinion, what did they think, we elaborated all tools together.” (Interview 21, nurse, leader of the quality improvement team, non-profit hospital). |
| 33 | “Working in a group is interesting because when one gets discouraged, the other gets encouraged, it is not a lonely work. The idea of building a quality improvement team is interesting because of that, because not everyone will be in the same mood all the time, but at one point, everyone has the same desire to see that change happen.” (Interview 1, physician, leader of the quality improvement team, non-profit hospital). |
| 34 | “We separated the group into teams, named them ventilator-associated pneumonia and urinary infection teams, we chose among the groups of technicians and nurses those who were group leaders or those who were better at assisting, those who were concerned with infection control.” (Interview 12, nurse, member of the quality improvement team, public hospital). |
| 35 | “What made it easier was the unity of the team. What I did in the project was to include the whole team, assemble teams, such as urinary infection, ventilator-associated pneumonia, and bloodstream infection teams. Each one with a team, we made an identification bottom, a yellow one for the urinary infection team, and a red one for the bloodstream infection team. That motivated them, and every month they presented the results in front of the organizational board, which influenced them, they discussed what could be improved or not.” (Interview 13, nurse, leader of the quality improvement team, public hospital). |
| **Theme: learning in practice** | |
| 36 | “In the hospital, a sequence of collaborative projects has been implemented, so we have a quality improvement team in the hospital that already existed before starting this project. I think the opportunity to gain more knowledge and bring changes in routines is valid, we can see during this period how it makes a difference for the hospital future.” (Interview 1, physician, leader of the quality improvement team, non-profit hospital). |
| 37 | “Education related to the knowledge of techniques is important: the education focused on improvement, socialization, dispersion of this knowledge. Because if we are not careful to pass this daily, they are retained in two or three people in a group.” (Interview 1, physician leader of the quality improvement team, non-profit hospital). |
| 38 | The more knowledge, the better, even if they are just a care person, they understand what the PDSA is, that change is to test everything, and you have to test it slowly and see if it works, I think it is important, even to understand better and have commitment.” (Interview 12, nurse, member of the quality improvement team, public hospital). |
| 39 | “I never had any contact with this type of experience and project, but it was important, it was enlightening and enriching because it made us look at the patient, look at care in a more specific and detailed way.” (Interview 18, nurse, member of the healthcare team, non-profit hospital). |
| **Theme: understanding the reason for changes** | |
| 40 | “We realize that this varies a lot, some professionals are more motivated, can understand the purpose of the project and the process improvement. Others cannot, they think we are charging.” (Interview 14, nurse, member of the quality improvement team, public hospital). |
| 41 | “This was the first time that we had a project for quality, in which everyone was involved and excited, it was very good.” (Interview 8, physician, member of the quality improvement team, non-profit hospital). |
| 42 | “To achieve the purposes of the project, we exchanged experiences with other hospitals that were going through the same process, some with more ease and others with more difficulties in conducting and achieving the purposes.” (Interview 14, nurse, member of the quality improvement team, public hospital). |
