# Supplementary material for: Motivational drivers for health professionals in a large quality improvement collaborative project in Brazil: a qualitative study
Source: BMC Health Serv Res. 2024 Feb 9;24:183. doi: 10.1186/s12913-024-10678-w (PMC10854114; doi:10.1186/s12913-024-10678-w)
Supplement: Supplementary file 2 — Supplementary Material 2 [file 12913_2024_10678_MOESM2_ESM.docx]

Additional File 2 - Consolidated criteria for reporting qualitative research (COREQ): a 32-item checklist for interviews and focus groups.

Reference: Tong A, Sainsbury P, Craig J. Consolidated criteria for reporting qualitative research (COREQ): a 32-item checklist for interviews and focus groups. Int Qual Health Care.2007;19(6):349-357.

| Nº | Item | Guide questions/description | Reported on Page and Section |
| --- | --- | --- | --- |
| **Domain 1: Research team and reflexivity** | | | |
| *Personal Characteristics* | | | |
| 1 | Interviewer/facilitator | Which author/s conducted the interview or focus group? | Pg. 7 |
| 2 | Credentials | What were the researcher’s credentials? | Pg. 7 |
| 3 | Occupation | What was their occupation at the time of the study? | Pg. 7 |
| 4 | Gender | Was the researcher male or female? | Pg. 7 |
| 5 | Experience and training | What experience or training did the researcher have? | Pg. 7 |
| *Relationship with participants* | | | |
| 6 | Relationship established | Was a relationship established prior to study commencement? | Pg. 8 |
| 7 | Participant knowledge of the interviewer | What did the participants know about the researcher? | Pg. 8 |
| 8 | Interviewer characteristics | What characteristics were reported about the interviewer/facilitator? | Pg. 7 |
| **Domain 2: study design** | | | |
| *Theoretical framework* | | | |
| 9 | Methodological orientation and Theory | What methodological orientation was stated to underpin the study? | Pg. 6, 9 |
| *Participant selection* | | | |
| 10 | Sampling | How were participants selected? | Pg. 7, 8 |
| 11 | Method of approach | How were participants approached? | Pg. 8 |
| 12 | Sample size | How many participants were in the study? | Pg. 9 |
| 13 | Non-participation | How many people refused to participate or dropped out? Reasons? | Pg. 7 |
| *Setting* | | | |
| 14 | Setting of data collection | Where was the data collected? | Pg. 7 |
| 15 | Presence of non-participants | Was anyone else present besides the participants and researchers? | Pg. 8 |
| 16 | Description of sample | What are the important characteristics of the sample? | Pg. 7 |
| *Data collection* | | | |
| 17 | Interview guide | Were questions, prompts, guides provided by the authors? Was it pilot tested? | Pg. 8 |
| 18 | Repeat interviews | Were repeat interviews carried out? If yes, how many? | Pg. 8 |
| 19 | Audio/visual recording | Did the research use audio or visual recording to collect the data? | Pg. 8 |
| 20 | Field notes | Were field notes made during and/or after the interview or focus group? | Not reported |
| 21 | Duration | What was the duration of the interviews or focus group? | Pg. 8 |
| 22 | Data saturation | Was data saturation discussed? | Pg. 7 |
| 23 | Transcripts returned | Were transcripts returned to participants for comment and/or correction? | Pg. 9 |
| **Domain 3: analysis and findings** | | | |
| *Data analysis* | | | |
| 24 | Number of data coders | How many data coders coded the data? | Pg. 10 |
| 25 | Description of the coding tree | Did authors provide a description of the coding tree? | Pg. 9 |
| 26 | Derivation of themes | Were themes identified in advance or derived from the data? | Pg. 10 |
| 27 | Software | What software, if applicable, was used to manage the data? | Pg. 9 |
| 28 | Participant checking | Did participants provide feedback on the findings? | Not reported |
| *Reporting* | | | |
| 29 | Quotations presented | Were participant quotations presented to illustrate the themes / findings? Was each quotation identified? | Pg. 10-17 |
| 30 | Data and findings consistent | Was there consistency between the data presented and the findings? | Pg. 17-24 |
| 31 | Clarity of major themes | Were major themes clearly presented in the findings? | S1 |
| 32 | Clarity of minor themes | Is there a description of diverse cases or discussion of minor themes? | Pg. 17-24 |
